# Endocrine‐related adverse events associated with immune‐checkpoint inhibitors in patients with melanoma

**DOI:** 10.1002/cam4.2533

**Published:** 2019-09-13

**Authors:** Eva Kassi, Anna Angelousi, Nikolaos Asonitis, Panagiotis Diamantopoulos, Amalia Anastasopoulou, George Papaxoinis, Michalis Kokkinos, Ilias Giovanopoulos, Georgios Kyriakakis, Fotini Petychaki, Akrivi Savelli, Olga Benopoulou, Helen Gogas

**Affiliations:** ^1^ First Department of Internal Medicine Laiko Hospital National and Kapodistrian University of Athens Athens Greece; ^2^ Department of Biological Chemistry Medical School National and Kapodistrian University of Athens Athens Greece

**Keywords:** checkpoint inhibitor, endocrinopathies, hypophysitis, melanoma, thyroid

## Abstract

**Background:**

Immune‐checkpoint inhibitors have been shown to improve survival in melanoma patients, but can also trigger immune‐related endocrinopathies, especially hypophysitis and thyroid dysfunction.

**Methods:**

To assess the incidence and the spectrum of endocrinopathies in melanoma patients treated with immunotherapy a prospective observational study was conducted. Forty out of 339 patients, treated with immune‐checkpoint inhibitors, developed endocrinopathies. All patients had hormonal functional tests at screening (before the initiation of immunotherapy) and during follow‐up.

**Results:**

The total incidence of endocrinopathies was 11.8%, 13.4% due to anti‐PD1/PDL1, 5% due to anti‐CTLA4, and 18.5% due to sequential and/or combination treatment. Twenty‐one patients (6.2%) presented with isolated anterior hypophysitis, eleven (3.2%) with primary thyroid dysfunction and eight (2.4%) with both abnormalities. The most frequent anterior pituitary hormone deficiency was central adrenal insufficiency, followed by central hypothyroidism and hypogonadotrophic hypogonadism. None of the patients with corticotroph axis failure recovered during follow‐up. Endocrinopathies occurred after a median of 22 weeks (range: 4‐156) from treatment initiation. Of note, sequential and/or combination therapy with anti‐CTLA4 and anti‐PD1/anti‐PDL1 led to an almost threefold incidence of hypophysitis compared to either monotherapy. Only one of 120 patients receiving anti‐CTLA4 monotherapy developed primary hypothyroidism.

**Conclusions:**

Our cohort demonstrated an increased incidence of hypophysitis with anti‐PD1/anti‐PDL1 in contrast to the rarity of primary thyroid dysfunction with anti‐CTLA4 treatment. These results could be attributed to genetic/ethnic differences. Sequential treatment is, for the first time to our knowledge, reported to increase the risk of developing hypophysitis to a level as high as that of combination therapy.

## Graphical abstract

A prospective observational study was conducted to assess the incidence and the spectrum of endocrinopathies in 339 melanoma patients treated with immune checkpoint inhibitors. Sequential and/or combination therapy with anti‐CTLA4 and anti‐PD1/anti‐PDL1 led to an almost threefold incidence of hypophysitis compared to either monotherapy, while only one of 120 patients receiving anti‐CTLA4 monotherapy developed primary hypothyroidism.

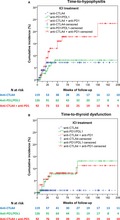

## 1 INTRODUCTION

Immunotherapy comprises a promising new cancer treatment which is designed to enhance a patient's immune system.^1^, ^2^ It includes the administration of monoclonal antibodies (mAbs) to proteins known as immune‐checkpoint inhibitors (ICIs)—cytotoxic T‐lymphocyte‐associated antigen‐4 (CTLA4), programmed cell death protein‐1 (PD1), and programmed death ligand 1 and 2 (PD‐L1 and PD‐L2), resulting in a derepression and/or reactivation of cytotoxic T‐cell function. Since 2001, the United States Food and Drug Administration (FDA) has approved several ICIs for the treatment of a broad spectrum of malignancies. Currently, seven ICIs are approved for the treatment of a number of advanced cancers.^3^ The anti‐PD1 Pembrolizumab and Nivolumab, and the anti‐CTLA4 ipilimumab were the first to be approved for the treatment of metastatic melanoma. ICIs enable a patient's immune system to attack cancer cells, although they can also attack certain healthy tissues. In other words, side effects associated with this therapy can implicate multiple organs.

Endocrinopathies affecting the pituitary and thyroid glands are emerging as some of the most common adverse events associated with immunotherapy and often the most symptomatic among these ICI‐related endocrinopathies (irEs).^4^ Meanwhile, apart from hypophysitis and thyroid dysfunction, insulin‐deficient diabetes mellitus and primary adrenal insufficiency have recently been reported as less frequent irEs due to ICI therapy and even more rarely hypoparathyroidism, however, all of them can be life‐threatening if not promptly recognized and treated.^5^, ^6^ Several observational studies and case series have reported that hypophysitis is mainly associated with anti‐CTLA4 therapy and thyroid dysfunction with anti‐PD1 therapy.^3^

The precise mechanisms of action underlying these endocrine irEs remain to be elucidated. In general, they are inflammatory in nature with the potential to affect multiple organs.^7^ While not occurring in all patients, they are believed to be an autoimmune response that results from the blocking of the normal immune regulatory pathways.^6^ Αccording to recent in vitro studies, pituitary gland expresses CTLA4, particularly in a subset of prolactin‐ and thyrotropin‐secreting cells. Thus, when these cells are blocked by the administration of a specific monoclonal antibody, inflammatory process is further activated leading to the deposition of C3 and C4 components in the pituitary cells. This is a potential mechanism to explain the pituitary toxicity observed in patients receiving ipilimumab.^7^ Interestingly, there is a correlation between overall patient survival and the incidence and severity of irEs.^8^, ^9^ The high survival rates in these cases might be due to the monitoring of patients for a longer period of time and the bias resulting from extended duration of symptomatic observation.^10^

To date, several retrospective and observational studies, including case reports and case series, have described the irEs resulting from immunotherapies. Nevertheless, an increasing list of ICIs has been approved for treatment of more types of cancer, and recently a new subclass of ICIs (anti PDL1 mAbs) was approved; in addition, new, more complicated therapeutic protocols, including combinations of ICI therapies are currently being used, while multiple lines of ICI treatment (sequential treatment) are being administered in patients with refractory cancer. Thus, the purpose of this prospective observational study was to describe the experience of a single‐center in Greece with patients with irEs, who had advanced melanoma and were treated with anti‐CTLA4 and anti‐PD1/anti‐PDL1, either as monotherapy, combination, or sequential treatment and with a long‐term follow‐up, in terms of incidence and specific characteristics (clinical, hormonal, and radiological.

## 2 METHODS

### 2.1 Subjects

In this prospective observational study patients with melanoma were enrolled. The primary endpoint of the study was to record the incidence of irEs with distinct type of ICI treatment, while secondary endpoints were to describe the epidemiology, presentation and clinical course of irEs. Inclusion criteria were a diagnosis of malignant melanoma and initiation of systemic treatment with ICI. Patients with cerebral metastases or undergoing cranial radiotherapy were excluded as well as patients receiving corticoid treatment at the moment of recruitment or had received corticoids recently (the last 2 months). Patients received ICIs with the following doses: Ipilimumab 3 mg/kg every 3 weeks, Pembrolizumab 2 mg/kg every 3 weeks, Nivolumab 3 mg/kg every 2 weeks, Atezolizumab 1200 mg and 1 mg/kg Nivolumab in combination with 3 mg/kg ipilimumab every 3 weeks. A small minority of nine patients (4.2% of all patients who received Ipilimumab as monotherapy or in combination with anti‐PD1) was treated with Ipilimumab 10 mg/kg.

Hormonal functional tests, such as cortisol, adrenocorticotropin hormone (ACTH), luteinizing hormone (LH), follicle stimulating hormone (FSH), estradiol, testosterone, and prolactin and thyroid stimulating hormone (TSH), as well as thyroid functional tests, including free thyroxine (fT4), free triiodothyronine (fT3) and anti‐ thyroid peroxidase (anti‐TPO) and anti‐thyroglobulin (anti‐Tg) antibodies as well as insulin growth factor‐1 (IGF‐1), were routinely measured before the initiation of the treatment, while cortisol, fT3, fT4, TSH and routine biochemical tests were performed before every other cycle, as well as if clinical signs and/or symptoms consistent with endocrinopathies developed. All measurements were performed in the morning before 9 am and analyzed in the same laboratory. Besides blood test analysis, pituitary magnetic resonance imaging (MRI) imaging and thyroid ultrasound were also performed if the patients presented with relevant clinical symptoms and abnormal hormonal functional tests.

Hypopituitarism was defined as low levels according to the laboratory normal ranges of the effector hormones (cortisol, fT4, sex hormone, and IGF‐1), with low or inappropriately normal pituitary hormone levels (ACTH and TSH), low gonadotrophins for age in postmenopausal women (LH, FSH), or low prolactin for gender. Functional hormonal assays were performed when needed: Synacthen test was performed in cases of suspicion of corticotrophin deficiency and gonadotroph axis deficiency with gonadotroph deficiency; however growth hormone (GH) deficiency was based only on low levels of IGF‐1 with low or inappropriate normal GH levels without performing any stimulating test. Patients were considered to have immunotherapy‐induced hypophysitis on the basis of biochemical hypopituitarism, clinical features, and/or radiologic findings. Primary thyroid dysfunction was defined as low levels of fT4 and/or fT3 with increased levels of TSH.

Patient data were prospectively registered through the medical records, hormonal tests, and radiological findings of 339 consecutive patients with melanoma, treated with ICIs between March 2014 and November 2018 at the Oncology Unit of the 1st Department of Internal Medicine at Laiko Hospital, Athens, Greece. Two investigators independently reviewed the patient data at the time of analysis. Patients presenting with irEs were selected and analyzed separately.

The study was conducted at Laiko Hospital in the 1st Department of Internal Medicine (National and Kapodistrian University of Athens, Greece), after institutional Human Ethics Review Committee approval according to the 1975 Declaration of Helsinki. Written informed consent was obtained from all participants.

### 2.2 Hormone assays

Hormone assays were performed using the following instruments with intra‐assay and inter‐assay coefficients of variability (CV) as follows: cortisol, TSH, fT4,fT3 and estradiol levels were measured using electrochemiluminescent bridging immunoassay (ECLIA) (Cobas 8000 e801, Hitachi, intra‐assay CV < 3.9% and inter‐assay CV < 3.8% for all hormones), ACTH, anti‐Tg, FSH, and LH were measured using chemiluminescent assay (Liaison, DiaSorin, intra‐assay CV 4.3%‐7.5% and inter‐assay 10%‐14.5%). IGF‐1 as well as GH levels were measured using radioimmunoassay (RIA).

### 2.3 Statistical analysis

Statistical analyses were performed using GraphPad Prism version 6 for Mac OS X (GraphPad Software) and Statistical Package for the Social Sciences for Windows version 22 (SPSS, Inc). Results are presented as median and interquartile range (IQR), or number and percentage. To compare two groups Student's *t* test for parametric continuous variables or the Mann‐Whitney U test for non‐parametric continuous variables were performed. To compare more than two groups, we used the Kruskal‐Wallis one‐way test. The chi‐square (*χ* ^2^ test) was used to compare categorical variables. The Kaplan‐Meier method was used to calculate the cumulative incidence of endocrine side effects at 52 weeks (approximately 1 year). All patients were followed for endocrine adverse events until the last date of follow‐up. After the development of an endocrine adverse event, all patients were followed for additional endocrine adverse events. Patients who did not develop an endocrine adverse event, were censored at their last date of follow‐up. Cox proportional hazard models were used to estimate the risk of developing an endocrine adverse event. All tests were two‐sided and a *P* < 0.05 was considered significant.

## 3 RESULTS

### 3.1 Patient characteristics

Out of a total of 339 patients with melanoma treated with ICIs, 40 patients (22 females) presented with irEs. The median age of the patients who presented with irEs was 61.3 years (Table 1). Τhe median total duration of treatment with ICIs was 5 months (range 2‐28), and the median total follow‐up was 15.95 months (range: 5‐57). Seventeen patients out of the 29 patients (59%) diagnosed with hypophysitis presented with fatigue, six with headaches (21%), and two (7%) with both symptoms, while four patients were asymptomatic (14%). All patients presenting with primary thyroid dysfunction complained of fatigue. MRI was performed in 26 patients presenting with pituitary axis insufficiency. Ten of them had several abnormalities of the pituitary gland: six had enlargement of the pituitary gland, three of them along with heterogeneity of the gland. Three other cases presented stack thickness. One patient presented a partial empty sella turcica. Only five of the patients had a second MRI during follow‐up; in three of them the size and consistency of the pituitary gland were normalized, in one patient a partial empty sella turcica had developed, and in the last patient the pituitary gland remained normal.

**Table 1:** Characteristics of the studied population treated with ICIs and the subgroup presenting with irEs

Characteristics	Total	PD1/PDL1 inhibitors	CTLA4 inhibitors	PD1/PDL1 and CTLA4 inhibitors (combined or sequentially)	*P*‐value
	N (%)	N (%)	N (%)	N (%)	
Total population treated with ICIs	339	127	120	92	
Age (median),mo	63.2	66.8	62.8	61.6	.175
Sex, F(%)	144 (42.5%)	44 (36.7%)	66 (52%)	34 (37%)	.024
Patients with irEs					
Age (median),months	61.3	68.2	61.3	60.1	.48
Sex, F(%)	22 (55%)	15 (88.2%)	2 (33.3%)	5 (29.4%)	.001
Total irEs	40 (11.8%)	17 (13.4%)	6 (5%)	17 (18.5%)	.02^a^, .3^b^, .002^c^
Isolated hypophysitis	21 (6.2%)	7 (5.5%)	5 (4.2%)	9 (9.8%)	.2^a^, .2^b^, .1^c^
Isolated primary thyroid dysfunction	11 (3.2%)	9 (7.1%)	0	2 (2.2%)	.003^a^, .1^b^, .1^c^
Both	8 (2.4%)	1 (0.8%)	1 (0.8%)	6 (6.5%)	1^a^, .017^b^, .022^c^

Abbreviations: F, female; ICIs, immune‐check point inhibitors; irEs, immune –related endocrinopathies.

^a^ Comparison of First line treatment with anti‐CTLA4 vs anti‐PD1/PDL1.

^b^ Comparison of First line treatment with anti‐PD1/PDL1 vs sequential/combination.

^c^ Comparison of First line treatment with anti‐CTLA4 vs sequential/combination.

Twenty‐four patients had PD1/PDL1 inhibitors treatment as first‐line immunotherapy Pembrolizumab, Nivolumab, or Atezolizumab), 11 patients had anti‐CTLA4 inhibitors (Ipilimumab) treatment and five patients had combination therapy of anti‐PD1and anti‐CTLA4 inhibitors (Ipilimumab and Nivolumab) (Tables 1 and 2). Of those treated with PD1/PDL1 inhibitors as first‐line seven switched to CTLA4 inhibitors as second‐line treatment, whereas of those treated with CTLA4 inhibitors as first‐line five switched to PD1/PDL1 inhibitors as second‐line treatment (sequential treatment) (Tables 1 and 2).

**Table 2:** Class and drug‐effect on the frequency of hypophysitis and primary thyroid dysfunction

Therapies	ICIs‐related endocrinopathies (irEs)		
	Isolated anterior pituitary insufficiency	Isolated primary thyroid dysfunction	Both
	N/Total (%)	N/Total (%)	N/Total (%)
Anti‐PD1/PDL1 monotherapy	7/17 (41.2%)^a^	9/17 (52.9%)^b^	1/17 (5.9%)^c^
Pembrolizumab	3/8	5/8	0/8
Nivolumab	2/7	4/7	1/7
Atezolizumab	2/2	0/2	0/2
Anti‐CTLA4 monotherapy	5/6 (83.3%)^a^	0/6 (0%)	1/6 (16.7%)^c^
Ipilimumab	5/6	0/6	1/6
Combination treatment	1/5 (20%)^a^	1/5 (20%)^b^	3/5 (60%)^c^
Ipilimumab + Nivolumab	1/5	1/5	3/5
Sequential treatment	8/12 (66.7%)^a^	1/12 (8.3%)^b^	3/12 (25%)^c^
Pembrolizumab ‐> Ipilimumab	5/6	0/6	1/6
Nivolumab ‐> Ipilimumab	0/1	0/1	1/1
Ipilimumab ‐> Pembrolizumab	3/4	0/4	1/4
Ipilimumab ‐> Nivolumab	0/1	1/1	0/1
Total	21/40 (53%)	11/40 (27.5%)	8/40 (20%)

Abbreviation: ICIs, immune‐check point inhibitors.

a Incidence of anti‐PD1/PDL1 and anti‐CTLA4–induced hypophysitis when they are used either as monotherapy or combination or sequential treatment.

b Incidence of anti‐PD1/PDL1 and anti‐CTLA4–induced primary thyroid dysfunction.

c Incidence of both anti‐PD1/PDL1 and anti‐CTLA4–induced hypophysitis and primary thyroid dysfunction when they are used either as monotherapy or combination or sequential treatment.

### 3.2 Incidence of endocrine‐related adverse events

The incidence of endocrine‐related adverse events in our study was 11.8% (40/339), with almost double incidence of hypophysitis compared to thyroid dysfunction (*P* = .01). The incidence of irEs due to anti‐PD1/PDL1 was 13.4%, due to anti‐CTLA4 5%, and due to sequential/combination therapy 18.5%. The difference in incidence between anti‐PD1/PDL1 treatment was statistically significantly different compared to the anti‐CTLA4 treatment (*P* = .02) (Table 1). The median time presentation of irEs after treatment initiation was 22 weeks (range: 4‐156). Twenty‐one out of 40 patients (53%) (10 women) presented with isolated anterior hypophysitis, 11 patients (27.5%) (nine women) with primary isolated hypothyroidism, and eight (20%) (three women) with both anterior hypophysitis and primary thyroid dysfunction. Endocrinopathies were numerically more frequent in women than in men (15.3% vs 9.2%, *P* = .092) although not statistically significant. Isolated anterior hypophysitis showed no difference between sexes (52% in men compared to 48% in women, *P* = .654), whereas primary thyroid dysfunction was more frequent among women (82%) compared to men (18%) (*P* = .011). Combined hypophysitis and thyroid dysfunction also showed no difference between sexes (62.5% in men vs 37.5% in women, *P* = 1).

### 3.3 Anterior hypophysitis related to ICIs

Twenty‐nine patients (8.6%) in total presented with hypophystitis, either isolated or in combination with primary thyroid dysfunction. Twenty‐one out of the total 29 patients (72%) who presented with anterior hypophysitis had insufficiency of the corticotroph axis, 7/29 (24%) of the gonadotroph axis, 4/29 (14%) of the somatotroph axis, and 7/29 (24%) of the thyrotroph axis (Table 3). It is of note that of the above 29 patients, 3/29 (10%) had deficiency of the corticotroph, the somatotroph, and the gonadotroph axis, 3/29 (10%) had insufficiency of the corticotroph and thyrotroph axis, and 6/29 (20%) had insufficiency of both the corticotroph and gonadotroph axis. The median time for the development of pituitary insufficiency after treatment initiation was 22 (range: 4‐156) weeks. All except one patient with thyrotroph axis deficiency recovered after a median of 8 months’ follow‐up (subsequent to interruption of immunotherapy). By contrast, none of the patients with corticotroph axis deficiency and only one patient with gonadotroph axis deficiency recovered during the final follow‐up (Table 3). Five out of the seven patients who recovered had been treated with monotherapy, whereas the remaining two had combination therapy with anti‐PD1 and anti‐CTLA4.

**Table 3:** Anterior hypophysitis axes deficiency

Axes deficiency	Anterior hypophysitis	Recovery after 15 mo of follow‐up
	N (%)	N (%)
Corticotroph	21 (72)	0 (0)
Thyreotroph	7 (24)	6 (86)
Somatotroph	4 (14)	NR
Gonadotroph	7 (24)	1 (14)
Lactotroph	2 (7)	NR
Panhypopituitarism	3 (10)	—
Corticotroph + Thyreotroph	3 (10)	—
Corticotroph + Gonadotroph	6 (20)	—
Total	29 (100)	7 (24)

Abbreviation: NR, not recorded.

### 3.4 Primary thyroid dysfunction related to ICIs

Nineteen patients (5.6%) in total presented with primary thyroid dysfunction; 11 patients out of the 40 (27.5%) presented with isolated primary thyroid dysfunction (all had hypothyroidism), while eight patients presented with both anterior hypophysitis and primary thyroid dysfunction (four of them presented with transient thyrotoxicosis due to destructive thyroiditis and four with hypothyroidism). Four out of these 11 patients had positive antithyroid antibodies (anti‐TPO). All patients with hypothyroidism received replacement therapy. The median time until primary thyroid dysfunction diagnosis was 20.5 (4‐108) weeks after treatment initiation. All but one patient with primary thyroid dysfunction had multinodular or heterogenous thyroid parenchyma. Five cases recovered, three of them were treated with monotherapy, one with combination therapy, and the last had sequential treatment.

### 3.5 Endocrine adverse events related to class effect

The total incidence of endocrine adverse events due to anti‐PD1/PDL1 inhibitors was 13.4% vs 5% due to anti‐CTLA4 inhibitors as monotherapy. Specifically, 17 out of 127 patients who were treated with anti‐PD1/PDL1 inhibitors and six out of 120 who were treated with anti‐CTLA4 inhibitors presented with endocrine adverse events (Table 1). Median age did not differ between patients who were treated with anti‐PD1/PDL1 vs anti‐CTLA4 monotherapy, while women were more common in those treated only with PD1/PDL1 inhibitors. The incidence of endocrine adverse events due to sequential and/or combination therapy was 18.5% (17.6% with sequential, 20.8% with combination therapy).

The incidence of isolated hypophysitis due to anti‐PD1/PDL1 monotherapy was 5.5% (7/127) vs 4.2% (5/120) due to anti‐CTLA4 monotherapy and 9.8% (9/92) due to sequential and/or combination therapy with both anti‐CTLA4 and anti‐PD1 (Table 1). The incidence of isolated primary thyroid dysfunction due to anti‐PD1/PDL1 monotherapy was 7.1%, whereas none of the six patients who received monotherapy with anti‐CTLA4 developed isolated primary thyroid dysfunction. The incidence of primary thyroid dysfunction due to sequential and/or combination therapy was 2.2%. Eight patients (20%) presented with both anterior hypophysitis and primary thyroid dysfunction; one of the latter patients was treated with anti‐PD1/PDL1 monotherapy, one with anti‐CTLA4 monotherapy, and six with combination and/or sequential therapy. The incidence of anterior hypophysitis in patients who received sequential/combination treatment was higher (16.3% in total, 16.2% with sequential, 16.7% with combination treatment) (15 out of 92 patients) compared to primary thyroid dysfunction (eight out of 92 patients) (8.7% in total, 5.9% with sequential, 16.7% with combination treatment) (Table 1).

Also, patients who received Ipilimumab 10 mg/kg as monotherapy or in combination with anti‐PD1 agents did not present statistically higher incidence of endocrine adverse effects, although their number was too small for definitive conclusions. More specifically endocrinopathies occurred in 2/9 patients with high‐dose Ipilimumab (22.2% vs 11.5% in the rest of patients, *P* = .288), hypophysitis in 2/9 patients with high‐dose Ipilimumab (22.2% vs 8.2% in the rest of patients, *P* = .175), and thyroid dysfunction in 1/9 patients with high‐dose Ipilimumab (11.1% vs 5.5% in the rest of patients, *P* = .409).

### 3.6 Risk of developing endocrine adverse events related to ICIs

**Figure 1:**
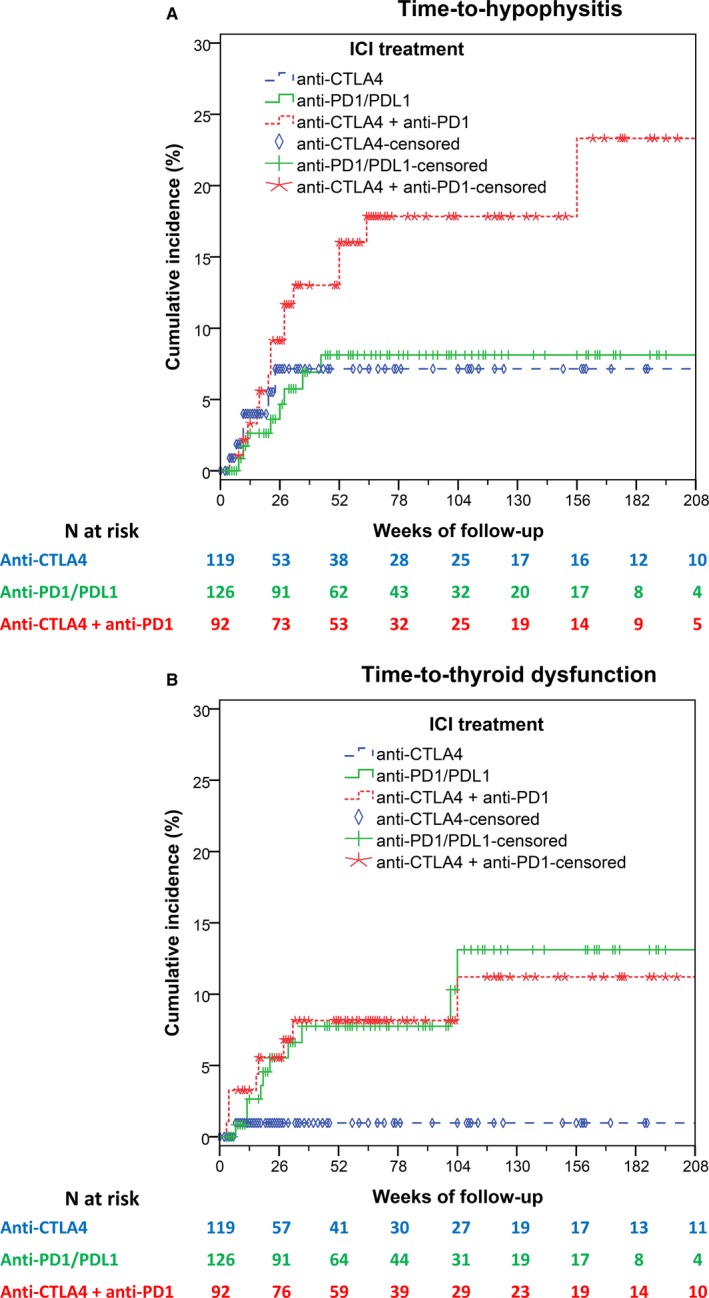
Cumulative incidence of hypophysitis (A) and thyroid dysfunction (B)

As shown in Figure 1A, sequential and/or combined treatment with both PD1/PDL1 and CTLA4 inhibitors as compared to monotherapy with PD1/PDL1 or CTLA4 inhibitors was associated with a higher risk of hypophysitis (hazard ratio [HR] 2.27, 95% confidence intervals [CI] 1.09‐4.70, *P* = .028) with a 52‐week cumulative incidence of 16% for patients treated with sequential and/or combination treatment, 8% for patients treated only with anti‐PD1/PDL1 monotherapy and 7% for patients treated only with anti‐CTLA4 monotherapy. Also, as shown in Figure 1B, treatment with PD1/PDL1 inhibitors (as monotherapy or sequentially or in combination with CTLA4 inhibitors) as compared to anti‐CTLA4 monotherapy was associated with a higher risk of thyroid dysfunction (HR 7.54, 95%CI 1.00‐56.55, *P* = .049) with a 52‐week cumulative incidence of 8% for both patients treated with sequential and/or combination treatment and those treated only with anti‐PD1/PDL1 monotherapy and 1% for patients treated only with anti‐CTLA4 monotherapy. The above associations remained significant even when adjusted for age and gender (Table 4).

**Table 4:** Multivariate Cox proportional hazard models demonstrating the significance of ICI treatment as risk factor for hypophysitis and thyroid dysfunction independent of age and gender

Cofactors for hypophysitis	Values	HR	95% CI	*P*‐value
Age	Advancing	0.99	0.97‐1.02	.528
Gender	Male vs Female	0.92	0.44‐1.91	.815
ICI treatment	Sequential and/or combination vs Monotherapy	2.27	1.09‐4.70	.028

Cofactors for thyroid dysfunction	Values	HR	95% CI	*P*‐value
Age (y)	Advancing	0.98	0.95‐1.02	.338
Gender	Male vs Female	0.54	0.21‐1.38	.198
ICI treatment	Anti‐PD1/PDL1 ± antiCTLA4 vs only anti‐CTLA4 treatment	7.54	1.00‐56.55	.049

Abbreviations: CI, confidence intervals; F, female; HR, hazard ratio; ICI, immune‐check point inhibitors.

## 4 DISCUSSION

Because of the ever more widespread use of ICIs for the treatment of various cancers, irEs are becoming more prevalent, making essential the early recognition of the disorders and prompt treatment. Herein, we report a prospective study of a large number of patients (n = 339) receiving ICIs for advanced melanoma as monotherapy, combination therapy, and sequential therapy in order to examine the incidence of the various endocrinopathies and the specific characteristics of them. We found that the total incidence of irEs was 11.8%, irrespective of the class of immune‐checkpoint inhibitor that was used. Among the entire group of 339 patients examined, the only irEs detected were thyroid dysfunction and hypophysitis, and, of note, the incidence of hypophysitis was almost double that of thyroid dysfunction, although the number of patients receiving each of the main two classes of ICIs was similar.

It is well‐established that hypophysitis and thyroid dysfunction are the most common irEs in ICI‐treated patients.^4^, ^11^, ^12^, ^13^, ^14^, ^15^ An interesting finding is that, although according to most studies PD1 and CTLA4 inhibitors produce endocrinopathies at almost similar frequencies when used as single agents,^16^ in this study, there was an almost threefold higher incidence among patients receiving anti‐PD1/PDL1 (13.4%) compared to those treated with anti‐CTLA4 (5%).^16^ This could be attributed to ethnic/race variations in polymorphisms of the *CTLA4* and *PD1/PDL1* genes, which have been described by Pincerati et al^17^ and are associated with increasing susceptibility to distinct autoimmune endocrinopathies.^18^, ^19^, ^20^, ^21^

Another interesting finding of our study is the much higher incidence of endocrine events with combination/sequential therapy compared to either anti‐PD1/PDL1 or anti‐CTLA4 monotherapy. Previous studies reported increased risk of multiple or single endocrinopathies in combination therapy compared to monotherapy.^22^, ^23^, ^24^ However, an incidence as high as 18.5% reported here, could be attributed—inter alia—to the long‐term follow‐up (median 15 months with a range of up to 57 months).

According to our data, there was a gender preference since more women developed irEs, although in most studies irEs appear to be more frequent in men.^3^, ^25^

The median time of diagnosis of irEs was 22 weeks post initiation of the immunotherapy. In previous reports, the median time to onset ranged between 4 and 18 weeks, with anti‐PD1 therapy related to earlier endocrine manifestations post initiation of therapy.^23^, ^26^, ^27^ However, most of the studies have a shorter follow‐up duration and a small number of patients while they have not included those receiving sequential therapy. It is also noteworthy that we had no severe (>grade 3) endocrine toxicities and no patient needing to permanently discontinue the immunotherapy.

In this study, we observed quite a high incidence (9%) of hypophysitis among patients treated with ICIs. In a meta‐analysis by Barroso‐Sousa et al^28^ among 6472 patients treated with any ICI, only 1.3% developed hypophysitis. We hypothesize that, possibly, among the factors contributing to this increased incidence are both increased awareness and close monitoring, as well as the long‐term follow‐up (3.2 years) of our patients; of interest, one patient developed hypophysitis 26 months post initiation of treatment.

It is worth noting that the risk of hypophysitis was higher among patients receiving anti‐PD1/PDL1 (incidence 6.3%) and lower among those subjects on anti‐CTLA4 (incidence 5.0%) monotherapy, compared to the data reported in the current literature. Indeed, in a meta‐analysis of 101 clinical studies (retrospective, prospective, and randomized trials) including 19922 patients, those treated with Ipilimumab developed hypophysitis at a rate of 5.6%, which was much higher than in anti‐PD1/PDL1 treated patients (0.5%‐1.5%).^24^, ^29^ Byun et al^4^ estimated that amongst 2017 Ipilimumab‐treated patients, 9.1% developed hypophysitis, while other large studies reported an incidence of Ipilimumab‐related hypophysitis equal to 13%, ranging from 1.5%‐17%.^9^, ^14^, ^30^ There is no apparent explanation for these divergent findings, which evidently need investigation; however, possible ethnic/race genetic variations could be hypothesized. Another potential explanation might be that cumulative experience with ICIs has increased the ability of oncologists to suspect irEs, especially hypophysitis, and proceed to endocrinology referral for formal diagnosis and proper management.

In line with previous studies, we found that sequential/combination therapy increased the incidence of hypophysitis to 16.3%. Larkin et al^31^ reported that the incidence of hypophysitis was 7.6% among 314 patients treated with combined therapy, while in two smaller studies by Wolchok et al^32^ and Postow et al,^33^ the incidence was 3.7% and 11.5%, respectively.

We included 68 patients receiving sequential therapy, either anti‐CTLA4 followed by anti‐PD1/PDL1 or the reverse. Eleven of them (16.2%) developed hypophysitis during the second‐line treatment. The increased incidence was irrespective of the class of ICIs given as first treatment. There are no data regarding endocrine adverse events during sequential therapy and, to our knowledge, the above‐mentioned increased risk of developing hypophysitis is herein reported for the first time and warrants further investigation. Interestingly, Das et al^34^ showed that blockade of either anti‐CTLA4 or anti‐PD1 alone leads to distinct genomic and functional signatures in purified human T cells and monocytes when compared to the combination of the two. This different immune response pattern could be responsible for the increased incidence of endocrine adverse events during combination as well as sequential therapy.

The most common symptom of hypophysitis was fatigue (59%). This is in line with previous studies that reported an incidence of 59%‐73%.^25^, ^35^, ^36^ Headache was less commonly described (21%), although two studies reported > 80% of patients experiencing this symptom.^35^, ^36^

None of the 26 patients recovered from central hypoadrenalism during a median follow‐up of 15 months, as likewise reported in previous studies,^22^, ^30^, ^35^, ^36^ while recovery from central hypothyroidism occurred in almost all patients. Similarly, to our results, Albarel et al^35^ described resolution of central hypothyroidism in 11/13 patients, while Scott et al^22^ reported the same in 0/10 patients. Of note, Min et al^30^ reported recovery in 14 of 22 patients in a median of 10 weeks.

MRI pituitary was performed in 26 out of 29 patients who developed hypophysitis, and abnormal radiographic pituitary findings were observed in 38% of patients. Most studies^25^, ^35^, ^36^ have reported a higher prevalence of radiological findings ranging from 75%‐100%; however, they all included fewer patients (n = 7‐17). Since the pituitary MRI was not performed as a screening test but due to clinical manifestations or biochemical findings, we cannot know if the abnormal radiographic features preceded the clinical diagnosis. Of note, the typical enlargement of pituitary and thickening of the stalk was described in only half of our patients who underwent MRI. Since radiological findings may precede the clinical manifestations by several weeks, while the pituitary enlargement can be resolved in less than two weeks,^36^ we hypothesize that an enlargement of the hypophysis – which was missed—preceded the appearance of the empty sella turcica.

Thyroid dysfunction developed in 5.6% patients receiving ICIs, which is a considerably lower incidence than that reported in most studies. Only one patient treated with Ipilimumab as monotherapy experienced thyroid adverse events: this is of great interest since we included a large number of patients receiving Ipilimumab. The combined therapy increased the incidence of thyroid disease, as has been previously reported. For the first time, it is reported that sequential therapy— irrespective of the class of ICIs used as first‐line treatment—did not increase further, compared to combined therapy, the incidence of thyroid disease. In contrast to our results, Scott et al^22^ reported a substantially higher incidence of thyroid dysfunction (14%) among patients receiving ICIs, with a prevalence of anti‐CTLA4 treated subjects. In a large study by Morganstein et al,^37^ the incidence of thyroid disease with Ipilimumab monotherapy was 23%, with anti‐PD1 monotherapy it was 39%, and with combination therapy it was 50%.

It should be noted that three patients (out of 19) developed hypothyroidism as late as 22‐24 months post initiation of treatment. This finding highlights the necessity for long‐term clinical and biochemical evaluation of patients treated with ICIs. Interestingly, only four patients developed thyrotoxicosis due to destructive thyroiditis, while no case of Graves’ disease was recorded. Lee et al^38^ studying retrospectively 45 patients with ICIs‐induced thyroid disorder found that 78% of patients developed thyrotoxicosis and only 22% exhibited hypothyroidism as a primary dysfunction.

The strengths of our study include the large number of patients (gender balanced) receiving anti‐PD1/anti‐PDL1, anti‐CTLA4, combined or sequential therapy, the close clinical and biochemical monitoring of the patients, and the long‐term follow‐up. Moreover, all the patients with endocrinopathies were referred to an endocrinologist. An important weakness of the study is that we cannot provide an apparent explanation for the vast differences in the rate of irEs between our study and the literature.

According to the data of our single‐center prospective observational study, irEs are quite common among patients receiving ICIs. In contrast to previous studies, we found an increased incidence of hypophysitis with anti‐PD1/anti‐PDL1 and a rarity of primary thyroid dysfunction with anti‐CTLA4 treatment, which could be attributed to genetic/ethnic variations. Sequential therapy, which is increasingly becoming common practice in order to prolong overall survival, is, for the first time to our knowledge, reported to increase the risk of developing hypophysitis as high as that associated with the combined therapy. Finally, our results highlight the need for long‐term follow‐up in order to achieve timely diagnosis and administer appropriate treatment, thereby avoiding the discontinuation of immunotherapy.
